# Redefining the active site: when the support takes centre stage

**DOI:** 10.1093/nsr/nwag264

**Published:** 2026-05-09

**Authors:** Leonardo da Silva Sousa, Andrew M Beale

**Affiliations:** Department of Chemistry, University College London, UK; UK Catalysis Hub, Research Complex at Harwell, Rutherford Appleton Laboratory, UK; Department of Chemistry, University College London, UK; UK Catalysis Hub, Research Complex at Harwell, Rutherford Appleton Laboratory, UK

Heterogeneous catalysis can be seen as the choreographing of atoms and molecules, where the catalyst’s surface acts as the stage. Reactant molecules arrive, break apart, rearrange, and recombine into new products, but only if every step happens in the right place and at the right time; reaction intermediates must find each other and react in the correct sequence to achieve selectivity to the desired product. However, with developments in measurement and detection it is becoming increasingly evident that subtleties in the way the ‘stage’ is assembled have a significant impact on the behaviour of the actors; the catalyst and especially its surface have been long known not be fixed objects, but something that constantly responds to their surroundings, evolving in response to their environment. Few reactions illustrate this better than ammonia synthesis. Nitrogen, with its triple bond, resists activation, while hydrogen must be split and delivered with precision. The catalyst must manage both, and it does so while continuously adapting: atoms shift, defects form and disappear, and the surface evolves dynamically.

**Figure 1.**
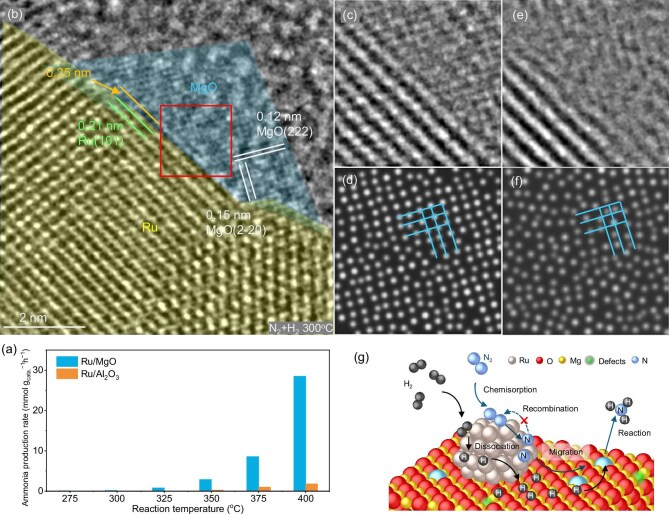
(a) Reaction performance of Ru/MgO and Ru/Al_2_O_3_ in ammonia synthesis reaction. *In situ* HRTEM images of (b) Ru/MgO heated in N_2_ and H_2_ at 300°C for 0 s and (c) region between Ru and MgO highlighted in the square in panel (b). (d) Contrast-intensified fast Fourier transformed (FFT)-filtered image of panel (c). (e) *In situ* HRTEM images of the same region as panel (c) after exposure to N_2_ and H_2_ at 300°C for 120 s. (f) The FFT-filtered image for panel (e). (g) The alternative pathway via Mg–N* for ammonia synthesis on Ru/MgO. Adapted with permission from ref. [1].

In this context, the work entitled ‘Stabilizing active N species on support for enhancing ammonia synthesis’ by Wang *et al.* [1] provides a striking window into how such dynamics unfold. The authors combine state-of-the-art environmental transmission electron microscopy with electron energy loss spectroscopy to probe Ru-based catalysts under reaction conditions. They compare 5 wt.% Ru/MgO and Ru/Al_2_O_3_ systems with similar Ru particle sizes (∼3 nm) and electronic states, yet markedly different catalytic performances, with Ru/MgO showing much higher ammonia synthesis rates, as shown in Fig. 1a. Through *in situ* environmental transmission electron microscopy (ETEM) (Fig. 1b–f), they observe structural changes at the metal–support interface, including the partial amorphization of MgO near Ru particles under H_2_ and N_2_ atmospheres. Electron energy loss spectroscopy (EELS) analysis reveals the formation of Mg–N species, providing direct evidence that nitrogen activated on Ru spills over to the support and is stabilized there. Complementary techniques such as diffuse reflectance infrared Fourier transform spectroscopy (DRIFTS) identify NH_x_ intermediates on the MgO surface, together with density functional theory (DFT) calculations showing favourable nitrogen incorporation into MgO defects. The study builds a coherent picture in which the metal particles and the support work in synergy (Fig. 1g), the reactant molecules are activated on the metallic Ru particle and they spillover onto the support, which plays a key role in stabilizing N* intermediates, creating a fine balance in which the support–intermediate interaction is strong enough to increase the Mg–N* residence time, and weak enough to allow for further reduction by H*.

The combination of high-resolution imaging, local spectroscopy, and theory delivers an unusually detailed description of the evolving catalyst structure under working conditions. Perhaps the most surprising insight is the role of the support. The authors show that a ‘nonreducible’ oxide like MgO can display behaviour typically associated with strong metal–support interactions typical for ‘reducible’ oxides, such as TiO_2_ and CeO_2_. Under reaction conditions, the support near the metal becomes disordered, almost amorphous, and this transformation is not a side effect but a key part of the chemistry. These newly formed defect sites can host reactive nitrogen species delivered through nitrogen spillover from the metal, thereby effectively pulling nitrogen intermediates away from metal sites and enabling subsequent reaction steps. This challenges a long-held view: that strong metal–support interactions require reducible oxides. Instead, it suggests that what truly matters is the ability of the support to restructure and create active environments as a consequence of significant defect generation. Amorphization of the support, often seen as inimical for catalytic performance, can be viewed as an extreme state capable of activating otherwise inert molecules. Indeed the observations made here are also similar to those previously demonstrated in the Ni/CeO_2_ system [2], where the hard-to-activate CO_2_ molecule is more readily converted when the CeO_2_ is seen to contain so many oxygen vacancies that the crystalline structure is lost. This leads to a support with a higher surface area, and subsequently, once the metal nanoparticles are also present, to more of the interfacial metal-support sites, which facilitate the formation of the intermediates that go on to form the desired products. A similar observation has been made for ZrO_2_-based catalysts for methanol synthesis, in which multiple reports [3] that obtained amorphous zirconia via low temperature synthesis had a higher initial catalytic activity that deactivated as the support crystallized under reaction conditions. Importantly these studies show again that the support often plays an active role in the catalytic process and that it does not need to be reducible in order to do this, rather that it is able to tolerate the formation of a number of defects particularly in the local vicinity of an active metal. Overall, Wang’s work is a further demonstration that in catalysis, when it comes to active sites, disorder can be just as important as order, and that even the most ‘inert’ materials can come alive under the right conditions.
